# ELDER-CENTERED RESEARCH METHODOLOGY: RESEARCH THAT DECOLONIZES AND INDIGENIZES

**DOI:** 10.1093/geroni/igac059.1513

**Published:** 2022-12-20

**Authors:** Maria Crouch, Steffi Kim, Zayla Asquith-Heinz, Elyse Decker, Nyche Andrew, Rosellen Rosich, Jordan Lewis

**Affiliations:** Yale School of Medicine, New Haven, Connecticut, United States; University of Minnesota, Minneapolis, Minnesota, United States; University of Minnesota Medical School, Minneapolis, Minnesota, United States; Swarthmore College, Swarthmore, Pennsylvania, United States; Yale University, New Haven, Connecticut, United States; University of Alaska Anchorage, Anchorage, Alaska, United States; University of Minnesota Medical School, Duluth, Minnesota, United States

## Abstract

Alaska Native (AN) Elders have historically been underrepresented in research. Innovative AN research posits that practice-based evidence is fundamental to culturally grounded, multifaceted methods. Semi-structured interviews were conducted with 34 AN Elders and 12 AN and non-Native caregivers in two studies exploring cultural understandings of memory and successful aging. The design and implementation of these studies employed Elders at every level of the research, ensuring cultural relevancy, outcomes, and dissemination activities. Findings reflect the benefits of engaging AN Elders in research and reveal methods for best practices: 1) creating Advisory councils; 2) identifying stakeholders 3) weaving together Elder and Western knowledge systems; and 4) the reciprocal nature of Elder engagement and wellbeing. This complimentary research builds on Elder-centric principles and lays the foundation for an Elder-centered research methodology that can be adapted and applied in other studies to encourage engagement of older adults in meaningful, restorative, and enculturated ways.

